# Density and distribution of lymphocytes in pretherapeutic rectal cancer and response to neoadjuvant therapy

**DOI:** 10.1093/gastro/goaa016

**Published:** 2020-06-12

**Authors:** Sicong Lai, Xiaoying Lou, Xinjuan Fan, Weipeng Sun, Yanhong Deng, Jianping Wang, Yan Huang, Ruoxu Dou

**Affiliations:** 1 Department of Colorectal Surgery, The Sixth Affiliated Hospital, Sun Yat-sen University, Guangzhou, Guangdong, P. R. China; 2 Guangdong Provincial Key Laboratory of Colorectal and Pelvic Floor Diseases, The Sixth Affiliated Hospital, Sun Yat-sen University, Guangzhou, Guangdong, P. R. China; 3 Department of Pathology, The Sixth Affiliated Hospital, Sun Yat-sen University, Guangzhou, Guangdong, P. R. China; 4 Department of Colorectal Surgery, The First Affiliated Hospital of Zhengzhou University, Zhengzhou, Henan, P. R. China; 5 Department of Medical Oncology, The Sixth Affiliated Hospital, Sun Yat-sen University, Guangzhou, Guangdong, P. R. China

**Keywords:** neoadjuvant therapy, tumor-infiltrating lymphocytes, therapeutic response, locally advanced rectal cancer

## Abstract

**Background:**

Lymphocytic density in rectal cancer has been reported to be associated with therapeutic response, but the role of the lymphocytic distribution pattern remains to be determined. This study aimed to evaluate the association between the distribution and density of lymphocytes in rectal-cancer tissue with tumor response to neoadjuvant therapy.

**Methods:**

We retrospectively analysed 134 patients with rectal cancer receiving neoadjuvant therapy within a prospectively maintained cohort. Pretherapeutic biopsy samples were stained with immunohistochemistry (CD4 and CD8). Densities of intratumoral periglandular lymphocytes (IPLs) and tumor-infiltrating lymphocytes (TILs) were assessed separately. Logistic-regression analysis was used to assess associations of lymphocyte densities with tumor regression grade (TRG), controlling for clinicopathological, molecular, and regimen features.

**Results:**

Compared with cases in the lowest quartile of CD8^+^ TILs, those in the highest quartile were significantly associated with better TRG (multivariate odds ratio, 0.23; 95% confidence interval, 0.07 to 0.76; *P *<* *0.001). In contrast, CD8^+^ IPLs, CD4^+^ IPLs, and CD4^+^ TILs were not significantly associated with TRG (*P *=* *0.033, 0.156, and 0.170, respectively). Sensitivity analyses detected no interaction between CD8^+^ TILs and regimen of neoadjuvant radiation (*P*_interaction_* *=* *0.831) or chemotherapy (*P*_interaction_* *=* *0.879) on TRG.

**Conclusions:**

Our data suggest that CD8^+^ TILs, but not IPLs, are independently associated with response to neoadjuvant therapy, regardless of the regimen of radiation or chemotherapy.

## Introduction

Neoadjuvant therapy followed by total mesorectal excision and adjuvant therapy has become the standard treatment for locally advanced rectal cancer [1, 2]. Neoadjuvant therapy has been shown to improve local control and reduce toxicity [3, 4]. However, response to neoadjuvant therapy varies, about translating into tumor downstaging in 60% and pathological complete response in 20% [5–7]. Pretherapeutic biopsies provide a precious resource for the study of predictive biomarkers to identify patients who benefit most.

The tumor immune microenvironment plays an important role in tumor biology and treatment. A high density of CD4^+^ and CD8^+^ tumor-infiltrating lymphocytes (TILs) within rectal-cancer tissue have shown better prognosis [8, 9]. The distribution of lymphocytes in rectal cancer also matters [10]. In addition, a recent study in breast cancer proves that lymphocytic density in pretherapeutic biopsy samples is predictive of response to neoadjuvant chemotherapy [11]. However, results vary among similar studies in colorectal cancer, possibly due to a lack of definition for cell type and distribution of lymphocytes [12–14]. Thus, the association of type, density, and location of lymphocytes with the therapeutic response to neoadjuvant therapy in locally advanced rectal cancer remains to be elucidated.

In this study, we stained pretherapeutic biopsy samples with immunohistochemistry (CD4 and CD8) and examined intratumoral periglandular lymphocytes (IPLs) and TILs in relation to post-treatment tumor regression grade (TRG).

## Method

### Study population

We retrospectively analysed 134 patients with locally advanced rectal cancer who had received neoadjuvant therapy followed by curative surgery in a prospectively maintained cohort of the Sixth Affiliated Hospital, Sun Yat-sen University (Guangzhou, China) between October 2010 and June 2016. All cases were rectal adenocarcinoma confirmed by pathology, with T3/T4 or N1 disease determined by pretherapeutic computed tomography or magnetic resonance imaging. All patients received neoadjuvant chemotherapy or chemoradiotherapy. Chemotherapy regimens included single agent (fluorouracil), duplet (FOLFOX), or triplet (FOLFOXIRI). The dosage of radiation was 45–50.4 Gy in 23–25 fractions. Patients with mucinous adenocarcinoma or recurrent rectal cancer and patients who did not receive protectomy were excluded (Supplementary Figure 1).

### Analysis of lymphocyte distribution and density in tumors

Previous studies have defined four components of lymphocyte reaction, including peritumoral lymphocytic reaction, Crohn's-like lymphoid reaction, IPL, and TIL [15]. Limited by the size and depth of pretherapeutic biopsy samples, peritumoral lymphocytic reaction and Crohn's-like lymphoid reaction could not be evaluated in most cases. Therefore, we investigated the other two components: IPL, defined as lymphocyte in tumor stroma within tumor mass; and TIL, defined as lymphocyte on top of cancer cells.

**Figure 1.**
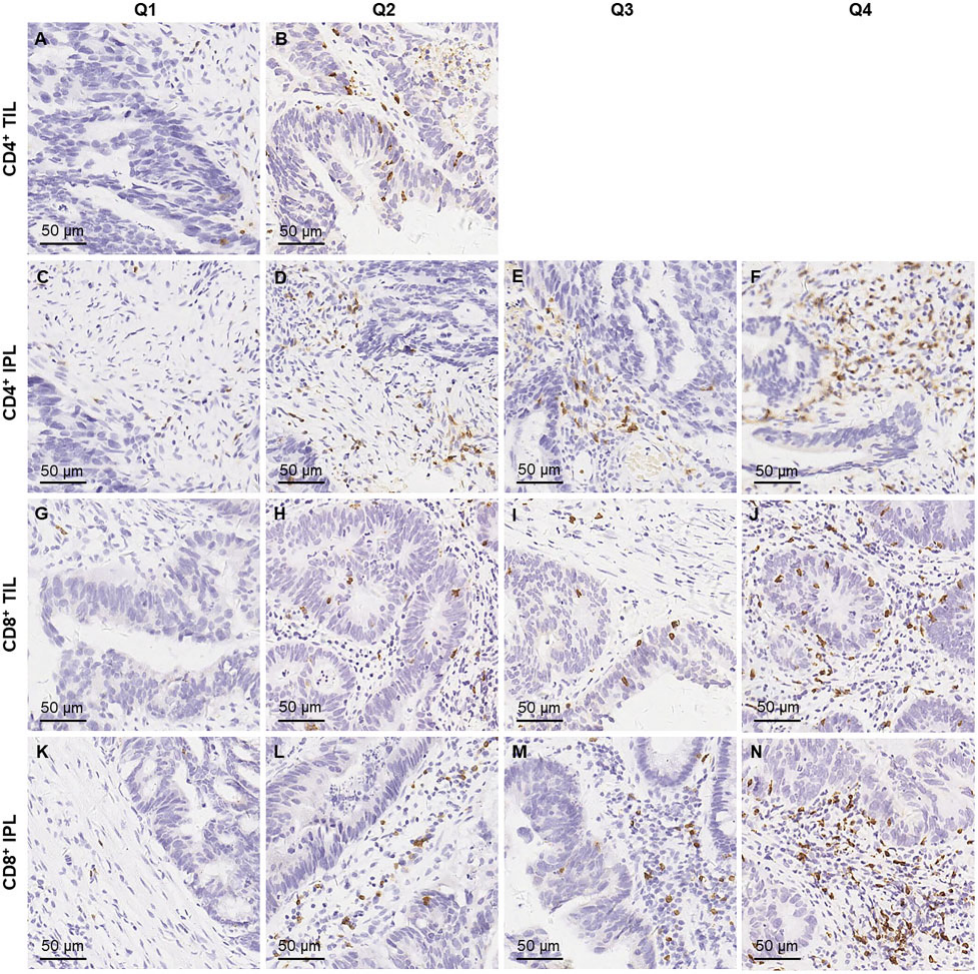
Classification of densities of TILs and IPLs, stained with immunohistochemistry of CD4 and CD8. (A, B) Two levels of CD4^+^ TILs, consisting of Q1, 0/mm^2^; and Q2, >0/mm^2^. (C–F) Quartiles of CD4^+^ IPLs, consisting of Q1, ≤25/mm^2^; Q2, >25 and ≤52/mm^2^; Q3, >52 and ≤110/mm^2^; and Q4, >110/mm^2^. (G–J) Quartiles of CD8^+^ TILs, consisting of Q1, ≤2/mm^2^; Q2, >2 and ≤5/mm^2^; Q3, >5 and ≤10/mm^2^; and Q4, >10/mm^2^. (K–N) Quartiles of CD8^+^ IPLs, consisting of Q1, ≤26/mm^2^; Q2, >26 and ≤41/mm^2^; Q3, >41 and ≤50/mm^2^; and Q4, >50/mm^2^. TIL, tumor infiltrating lymphocyte; IPL, intratumoral periglandular lymphocyte.

Tissue sections from all rectal-cancer cases were examined by two pathologists unaware of other data. For this study, slides were available for 134 cases for immunohistochemistry staining with CD4 and CD8 [CD4 (1:250, ZA0519; ZSGB-BIO, Beijing, China) and CD8 (1:500, ZA0508; ZSGB-BIO, Beijing, China)] (Supplementary Figure 1). CD4^+^ (Figure 1A–F) and CD8^+^ T-cells (Figure 1G–N) were counted at five random fields at 400× magnification and analysed in quantiles. Limited by the size of pretherapeutic biopsy samples, CD4^+^ and CD8^+^ T-cells were counted at three random fields for two cases.

### Analysis of tumor response to neoadjuvant therapy

TRG after neoadjuvant therapy was routinely evaluated according to the American Joint Committee on Cancer-Cancer Staging Manual, Eighth Edition: 0, no viable cancer cells (complete response); 1, single cells or rare small groups of cancer cells (near-complete response); 2, residual cancer with evident tumor regression, but more than single cells or rare small groups of cancer cells (partial response); and 3, extensive residual cancer with no evident tumor regression (poor or no response).

### Statistical analysis

None of the lymphocyte densities or their log-transformed values fit a normal distribution in the Kolmogorov–Smirnov test for normality (*P *<* *0.001). Thus, we used a statistical trend test across the ordinal quantiles of lymphocyte density as a continuous variable. To test the association between pretherapeutic lymphocyte density and tumor regression, we used a logistic-regression model to test the association of lymphocyte density (an ordinal quantile predictor variable) with tumor regression grade (a binary outcome variable, TRG 0–1 vs 2–3). Considering multiple comparisons (TIL and IPL as stained by CD4^+^ and CD8^+^ antibodies), α level was adjusted to 0.0125 (=* *0.05/4) by simple Bonferroni correction. To control for confounding factors, the multivariate logistic-regression model initially included age (continuous), sex, tumor location (upper vs middle vs lower rectum), clinical T and N stage, mismatch repair (MMR) status (deficient vs proficient), regiment of neoadjuvant chemotherapy (single agent vs doublet vs triplet), and neoadjuvant radiotherapy (yes vs no). A backward stepwise elimination with a threshold of *P *=* *0.05 was used to select covariates in the final model. For cases missing any categorical covariate, such as tumor differentiation (5%) and MMR status (11%), we included those cases in a majority category of the covariate.

All other analyses for clinical, pathological, and therapeutic associations were secondary exploratory analyses and we adjusted the two-sided α level to 0.005 (=* *0.05/10) by simple Bonferroni correction for multiple comparisons. To assess associations between the quantiles of lymphocyte density and categorical data, the chi-square test was performed. To compare continuous data, an analysis of variance assuming equal variances was performed. We used the SPSS program (Version 20.0, IBM, Cary, NC) for all statistical analyses. All *P-*values were two-sided.

## Results

### Density of lymphocytes in rectal-cancer tissue

For quality control, the Spearman correlation coefficient between the two evaluators was 0.95 on TRG (binary 0–1 vs 2–3), 0.85 on CD4^+^ IPLs (*P *<* *0.001), 0.82 on CD4^+^ TILs, (*P *<* *0.001), 0.83 on CD8^+^ IPLs (*P *<* *0.001), and 0.82 on CD8^+^ TILs (*P *<* *0.001).

We measured T-cell densities by CD4 and CD8 staining in 134 cases. Clinical, pathological, and molecular characteristics of the measured cases are shown according to densities of CD4^+^ (Table 1) and CD8^+^ T-cells (Table 2). Because there was no CD4^+^ TIL in more than 75% of cases, only two levels were presented for CD4^+^ TIL (Q1, none of CD4^+^ TIL; Q2, at least one or more CD4^+^ TILs). After adjusting for multiple comparisons, none of the characteristics was statistically significantly associated with the density of CD4^+^ and CD8^+^ T-cells.

**Table 1.** Clinicopathological characteristics and CD4^+^ T-cell densities in 134 colorectal-cancer tissues

Characteristic	Total	CD4^+^ TIL			CD4^+^ IPL				
		Q1	Q2	*P* *	Q1	Q2	Q3	Q4	*P* *
Sex				0.195					0.186
Male	94 (70.1)	85 (72.0)	9 (56.3)		24 (68.6)	25 (78.1)	20 (57.1)	25 (78.1)	
Female	40 (29.9)	33 (28.0)	7 (43.7)		11 (31.4)	7 (21.9)	15 (42.9)	7 (21.9)	
Age, years				0.593					0.527
<60	84 (62.7)	73 (61.9)	11 (68.8)		24 (68.6)	22 (68.8)	19 (54.3)	19 (59.4)	
≥60	50 (37.3)	45 (38.1)	5 (31.2)		11 (31.4)	10 (31.2)	16 (45.7)	13 (40.6)	
Tumor differentiation				0.376					0.658
Well	52 (38.8)	47 (39.8)	5 (31.3)		13 (37.1)	12 (37.5)	12 (34.3)	15 (46.9)	
Moderate	74 (55.2)	63 (53.4)	11 (68.7)		18 (51.4)	18 (56.3)	22 (62.9)	16 (50.0)	
Poor	8 (6.0)	8 (6.8)	0 (0)		4 (11.5)	2 (6.2)	1 (2.8)	1 (3.1)	
T category				0.762					0.902
T3	99 (73.9)	88 (74.6)	11 (68.7)		27 (77.1)	21 (65.6)	27 (77.1)	24 (75.0)	
T4	35 (26.1)	30 (25.4)	5 (31.3)		8 (22.9)	11 (34.4)	8 (22.9)	8 (25.0)	
N category				0.525					0.772
0	107 (79.9)	93 (78.8)	14 (87.5)		28 (80.0)	27 (84.4)	26 (74.3)	26 (81.3)	
1	27 (20.1)	25 (21.2)	2 (12.5)		7 (20.0)	5 (15.6)	9 (25.7)	6 (18.7)	
Tumor location				0.790					0.933
Upper (10–15 cm)	12 (8.9)	10 (8.5)	2 (12.5)		2 (5.7)	4 (12.5)	4 (11.4)	2 (6.2)	
Middle (5–10 cm)	65 (48.5)	57 (48.3)	8 (50.0)		18 (51.4)	16 (50.0)	16 (45.7)	15 (46.9)	
Low (0–5 cm)	57 (42.6)	51 (43.2)	6 (37.5)		15 (42.9)	12 (37.5)	15 (42.9)	15 (46.9)	
MMR				0.100					0.670
dMMR	10 (7.5)	7 (5.9)	3 (18.8)		2 (5.7)	2 (6.3)	2 (5.7)	4 (12.5)	
pMMR	124 (92.5)	111 (94.1)	13 (81.2)		33 (94.3)	30 (93.7)	33 (94.3)	28 (87.5)	

All values are presented as numbers of patients followed by percentages in parentheses.

* To assess associations between the ordinal categories of lymphocyte densities with categorical data, the chi-square test was performed. To compare continuous variables, the analysis of variance was performed. We adjusted two-sided α level to 0.007 (= 0.05/7) by simple Bonferroni correction.

TIL, tumor-infiltrating lymphocyte; IPL, intratumoral periglandular lymphocyte; Q1 to Q4, quantile 1 (lowest) to quantile 4 (highest); MMR, mismatch repair; dMMR, deficient mismatch repair; pMMR, proficient mismatch repair.

**Table 2.** Clinicopathological characteristics and CD8^+^ T-cell densities in 134 colorectal-cancer tissues

Characteristic	Total	CD8^+^ TIL					CD8^+^ IPL				
		Q1	Q2	Q3	Q4	*P* *	Q1	Q2	Q3	Q4	*P* *
Sex						0.583					0.504
Male	94 (70.1)	26 (72.2)	34 (75.6)	17 (60.7)	17 (68.0)		26 (78.8)	33 (71.7)	14 (63.6)	21 (63.6)	
Female	40 (29.9)	10 (27.8)	11 (24.4)	11 (39.3)	8 (32.0)		7 (21.2)	13 (28.3)	8 (36.4)	12 (36.4)	
Age, years						0.160					0.502
<60	84 (62.7)	25 (69.4)	23 (51.1)	21 (75.0)	15 (60.0)		23 (69.7)	25 (54.3)	15 (68.2)	21 (63.6)	
≥60	50 (37.3)	11 (30.6)	22 (48.9)	7 (25.0)	10 (40.0)		10 (30.3)	21 (45.7)	7 (31.8)	12 (36.4)	
Tumor differentiation						0.375					0.025
Well	52 (38.8)	14 (38.9)	21 (46.7)	6 (21.4)	11 (44.0)		11 (33.3)	17 (37.0)	7 (31.8)	17 (51.5)	
Moderate	74 (55.2)	19 (52.8)	23 (51.1)	20 (71.4)	12 (48.0)		16 (48.5)	28 (60.9)	14 (63.6)	16 (48.5)	
Poor	8 (6.0)	3 (8.3)	1 (2.2)	2 (7.2)	2 (8.0)		6 (18.2)	1 (2.1)	1 (4.6)	0 (0)	
T stage						0.561					0.530
T3	99 (73.9)	26 (72.2)	36 (80.0)	17 (60.7)	20 (80.0)		24 (72.7)	36 (78.3)	14 (63.6)	25 (75.8)	
T4	35 (26.1)	10 (27.8)	9 (20.0)	11 (39.3)	5 (20.0)		9 (27.3)	10 (21.7)	8 (36.4)	8 (24.2)	
N stage						< 0.001					0.215
0	107 (79.9)	21 (58.3)	34 (75.6)	27 (96.4)	25 (100.0)		26 (78.8)	33 (71.7)	18 (81.8)	30 (90.9)	
1	27 (20.1)	15 (41.7)	11 (24.4)	1 (3.6)	0 (0)		7 (21.2)	13 (28.3)	4 (18.2)	3 (9.1)	
Tumor location						0.879					0.596
Upper (10–15 cm)	12 (8.9)	3 (8.3)	5 (11.1)	2 (7.2)	2 (8.0)		4 (12.1)	1 (2.2)	3 (13.6)	4 (12.1)	
Middle (5–10 cm)	65 (48.5)	18 (50.0)	19 (42.2)	13 (46.4)	15 (60.0)		14 (42.4)	24 (52.2)	10 (45.5)	17 (51.5)	
Low (0–5 cm)	57 (42.6)	15 (41.7)	21 (46.7)	13 (46.4)	8 (32.0)		15 (45.5)	21 (45.6)	9 (40.9)	12 (36.4)	
MMR						0.641					0.602
dMMR	10 (7.5)	2 (5.6)	4 (8.9)	1 (3.6)	3 (12.0)		2 (6.1)	2 (4.3)	2 (9.1)	4 (12.1)	
pMMR	124 (92.5)	34 (94.4)	41 (91.1)	27 (96.4)	22 (88.0)		31 (93.9)	44 (95.7)	20 (90.9)	29 (87.9)	

All values are presented as numbers of patients followed by percentages in parentheses.

* To assess associations between the ordinal categories of lymphocyte densities with categorical data, the chi-square test was performed. To compare continuous variables, the analysis of variance was performed. We adjusted two-sided α level to 0.007 (= 0.05/7) by simple Bonferroni correction. TIL, tumor-infiltrating lymphocyte; IPL, intratumoral periglandular lymphocyte; Q1 to Q4, quantile 1 (lowest) to quantile 4 (highest); MMR, mismatch repair; dMMR, deficient mismatch repair; pMMR, proficient mismatch repair.

### Density of lymphocytes and TRG in rectal-cancer tissue

We evaluated TRG after neoadjuvant therapy on 134 post-operative rectal-cancer tissues. Table 3 shows the distribution of cases according to the densities of CD4^+^ and CD8^+^ T-cells (quantiles) and TRG in rectal-cancer tissue.

**Table 3.** Density of CD4^+^ and CD8^+^ T-cells and TRG in rectal-cancer tissue

Lymphocyte	TIL						IPL					
density	Total	TRG0	TRG1	TRG2	TRG3	*P* *	Total	TRG0	TRG1	TRG2	TRG3	*P* *
CD4^+^						0.170						0.156
Q1	118	23 (19.5)	30 (25.4)	51 (43.2)	14 (11.9)		35	3 (8.6)	8 (22.8)	21 (60.0)	3 (8.6)	
Q2	16	1 (6.2)	3 (18.8)	10 (62.5)	2 (12.5)		32	6 (18.8)	9 (28.1)	10 (31.3)	7 (21.8)	
							35	7 (20.0)	11 (31.4)	13 (37.1)	4 (11.5)	
							32	8 (25.0)	5 (15.6)	17 (53.1)	2 (6.3)	
CD8^+^						<0.001						0.033
Q1	36	2 (5.6)	8 (22.2)	12 (33.3)	14 (38.9)		33	5 (15.2)	11 (33.3)	11 (33.3)	6 (18.2)	
Q2	45	3 (6.7)	5 (11.1)	35 (77.8)	2 (4.4)		46	3 (6.5)	7 (15.2)	29 (63.1)	7 (15.2)	
Q3	28	11 (39.3)	11 (39.3)	6 (21.4)	0		22	7 (31.8)	8 (36.4)	6 (27.3)	1 (4.5)	
Q4	25	8 (32.0)	9 (36.0)	8 (32.0)	0		33	9 (27.3)	7 (21.2)	15 (45.5)	2 (6.0)	

All values are presented as numbers of patients followed by percentages in parentheses.

* To compare categorical data between TRG degrees, the chi-square test was performed. We adjusted two-sided α level to 0.0125 (= 0.05/4) by simple Bonferroni correction.

TIL, tumor-infiltrating lymphocyte; IPL, intratumoral periglandular lymphocyte; TRG, tumor regression grade; Q1 to Q4, quantile 1 (lowest) to quantile 4 (highest).

We conducted univariate and multivariate logistic-regression analyses to assess the associations of density of CD4^+^ and CD8^+^ T-cells (Table 4) as an ordinal quartile predictor variable with TRG as a binary outcome variable (lower score suggests better response). CD8^+^ TIL density was significantly associated with TRG (multivariate *P *<* *0.001 with adjusted α level at 0.0125). Compared with cases in the lowest quartile of CD8^+^ TIL density, those in the highest quartile were more likely to have a better response to neoadjuvant therapy [multivariate odds ratio (OR), 0. 23; 95% confidence interval (CI), 0.07 to 0.76]. In contrast, densities of CD8^+^ IPLs, CD4^+^ IPLs, and CD4^+^ TILs were not significantly associated with TRG (*P *=* *0.033, 0.156, and 0.170, respectively).

**Table 4.** Clinicopathological characteristics, CD4^+^ and CD8^+^ T-cell densities, and TRG in 134 rectal-cancer tissues

Characteristic^a^	Univariate OR (95% CI)	*P* *	Multivariate OR (95% CI)	*P* *
Neoadjuvant radiation		<0.001		0.010
No	1 (Reference)		1 (Reference)	
Yes	0.24 (0.12–0.51)		0.31 (0.12–0.80)	
Regimen of chemotherapy		0.707		
Single agent	1 (Reference)			
Doublet	2.93 (0.98–8.80)			
Triplet	1.65 (0.47–5.77)			
Cycles of chemotherapy		<0.001		0.414
≤4	1 (Reference)		1 (Reference)	
>4	0.20 (0.09–0.41)		0.65 (0.23–1.82)	
CD4^+^ TIL density		0.140		
Q1	1 (Reference)			
Q2	2.45 (0.74–8.03)			
CD4^+^ IPL density		0.368		
Q1	1 (Reference)			
Q2	0.65 (0.24–1.70)			
Q3	0.78 (0.29–2.09)			
Q4	1.49 (0.55–4.07)			
CD8^+^ TIL density		<0.001		<0.001
Q1	1 (Reference)		1 (Reference)	
Q2	1.78 (0.62–5.12)		2.52 (0.80–7.92)	
Q3	0.10 (0.03–0.34)		0.18 (0.05–0.65)	
Q4	0.18 (0.06–0.55)		0.23 (0.07–0.76)	
CD8^+^ IPL density		0.232		
Q1	1 (Reference)			
Q2	0.44 (0.14–1.36)			
Q3	3.39 (1.27–9.01)			
Q4	1.00 (0.38–2.63)			

^a^ The multivariate Cox-regression model initially included sex, age, tumor differentiation, T stage, N stage, tumor location, mismatch repair, neoadjuvant radiation, regimen and cycles of chemotherapy, and T-cell densities. A backward stepwise elimination with a threshold of *P* < 0.05 was used to select variables in the final models.

* *P* was calculated by the linear-trend test across the ordinal categories as a continuous input variable in the logistic-regression model for TRG as a binary outcome variable. By simple Bonferroni correction, we adjusted the two-sided α level to 0.0125 (= 0.05/4) for the primary hypothesis testing for CD4^+^ and CD8^+^ T-cells and to 0.005 (=0.05/10) for all other exploratory covariates.

TRG, tumor regression grade; OR, odds ratio; CI, confidence interval; TIL, tumor-infiltrating lymphocyte; IPL, intratumoral periglandular lymphocyte; Q1 to Q4, quantile 1 (lowest) to quantile 4 (highest).

### Associations of clinicopathological characteristics and therapeutic approaches with TRG in rectal-cancer tissue

None of the pretherapeutic clinicopathological characteristics was significantly associated with TRG in univariate analyses (all *P *>* *0.05, Supplementary Table 1). Neoadjuvant radiation was significantly associated with better treatment response (multivariate *P *=* *0.010, with adjusted α level at 0.0125; Table 4). Subgroup analysis showed no significant interaction between neoadjuvant radiation or regimen of chemotherapy and CD8^+^ TILs on TRG (all *P*_interaction_* *>* *0.05; Table 5).

**Table 5.** Subgroup analysis of CD8^+^ TIL and TRG in 134 rectal-cancer tissues

Group	CD8^+^ TIL	*n*	Univariate OR (95% CI)	*P* *_trend_	Multivariate OR (95% CI)	*P* *_trend_	*P*_interaction_
Radiation							0.831
Yes	Q1	12	1 (Reference)	0.006	1 (Reference)	0.005	
	Q2	22	3.40 (0.75–15.3)		3.40 (0.61–19.0)		
	Q3	20	0.05 (0.005–0.53)		0.10 (0.008–1.33)		
	Q4	14	0.40 (0.079–2.02)		0.32 (0.04–2.26)		
No	Q1	24	1 (Reference)	0.005	1 (Reference)	0.004	
	Q2	23	1.33 (0.26–6.74)		1.23 (0.24–6.30)		
	Q3	8	0.33 (0.06–2.00)		0.38 (0.06–2.35)		
	Q4	11	0.11 (0.02–0.58)		0.11 (0.02–0.59)		
Regiment of chemotherapy							0.879
Single agent	Q1	5	1 (Reference)	0.059	1 (Reference)	0.035	
	Q2	16	10.0 (0.67–149)		9.85 (0.41–235)		
	Q3	3	N/A		N/A		
	Q4	7	0.27 (0.02–3.02)		0.48 (0.03–7.95)		
Doublet	Q1	24	1 (Reference)	0.001	1 (Reference)	0.002	
	Q2	23	1.54 (0.32–7.50)		0.52 (0.08–3.40)		
	Q3	21	0.13 (0.03–0.56)		0.10 (0.02–0.53)		
	Q4	15	0.15 (0.04–0.63)		0.16 (0.03–0.80)		
Triplet	Q1	7	1 (Reference)	0.287	1 (Reference)	0.227	
	Q2	6	2.00 (0.09–44.4)		1.10 (0.04–35.0)		
	Q3	4	0.40 (0.02–10.0)		0.26 (0.007–8.94)		
	Q4	3	0.33 (0.01–8.18)		0.21 (0.006–7.18)		

* The multivariate Cox-regression model initially included sex, age, tumor differentiation, T stage, N stage, tumor location, mismatch repair, neoadjuvant radiation, regimen and cycles of chemotherapy, and T-cell densities. A backward stepwise elimination with a threshold of *P* < 0.05 was used to select variables in the final models.

TIL, tumor-infiltrating lymphocyte; TRG, tumor regression grade; OR, odds ratio; CI, confidence interval; Q1 to Q4, quantile 1 (lowest) to quantile 4 (highest); N/A, not applicable.

## Discussion

We conducted this study to test the association between the distribution and density of CD4^+^ and CD8^+^ T-cells in rectal-cancer tissue with tumor response to neoadjuvant therapy. We found a positive association between the CD8^+^ TIL density in rectal-cancer tissue and treatment response.

Densities of various lymphocytes in tumor tissues, particularly those of CD8^+^ T-cells, have been reported to be associated with the clinical outcome of colorectal cancer [16]. Spatial studies of lymphocytic distribution further suggested a better prognostic value from tumor epithelial infiltration than from stromal infiltration [17]. Various patterns of lymphocytic distribution have been proposed [15]. A scoring system named immunoscore that integrates the density and distribution of CD3^+^ and CD8^+^ T-cells in tumor tissues has been proven to be a robust prognostic marker [10].

Beyond being prognostic, the density of lymphocytes in colorectal tissues might be associated with response after neoadjuvant therapy [14]. In biopsy samples of breast cancer before neoadjuvant therapy, the density of intratumoral, but not stromal, lymphocytes was an independent predictor for treatment response [11]. Such an association between the distribution of lymphocytes and the treatment response has been rarely investigated in colorectal cancer. A previous study with 93 cases showed that a high pretherapeutic CD8^+^/FOXP3^+^ intraepithelial lymphocyte ratio was associated with favorable tumor regression [13], which is consistent with our finding of a specific association between the density of CD8^+^ TIL and the treatment response. Approximately 80% patients with the lower CD8^+^ TIL density (Q1 or Q2) were graded TRG 2–3. Neoadjuvant therapy is unlikely to achieve a significant tumor response in them, but still causes adverse events. Thus, we propose future comparative studies on different approaches for these patients.

Penter *et al.* [18] found that expanded T-cell clones were predominantly CD8^+^. We found that the lack of CD4^+^ TILs limits the clinical significance and statistical power of CD4^+^ TIL comparison in this scenario. The likely elution of CD4^+^ TILs reflects an effect not only on subpopulations, but also on T-cell functioning within the remaining CD8^+^ cells, which can be studied with flow cytometry and T-cell receptor signaling on fresh biopsies.

Neoadjuvant radiation and chemotherapy have been reported to release neoantigen by killing tumor cells [19]. TILs have been proposed to synergize with therapies by facilitating a treatment-elicited immune response [20]. Radiation was associated with therapeutic response and remained independent after multivariate analysis. However, our sensitivity analyses detected no interaction between CD8^+^ TILs and traditional chemoradiotherapy.

Besides cytotoxic neoadjuvant chemotherapy, it would be interesting to investigate the interaction between TILs (especially CD8^+^ TILs) and neoadjuvant immune checkpoint blockade [21–24]. Studies have found that patients with a higher neoantigen load have elevated TILs and longer survival [25]. Moreover, TIL infiltrations have been associated with response to immune checkpoint blockade in the metastatic setting [26]. Nevertheless, evidence is lacking for neoadjuvant immune therapies.

One limitation of our study is the retrospective observational design. However, we investigated a pre-specified hypothesis in a large, consecutive cohort. In addition, the strong association between CD8^+^ TILs and therapeutic response remained in multivariate analysis, after adjusting for possible confounding factors including differentiation, T/N category, MMR, and neoadjuvant regimen. Another limitation is the size and depth of the pretherapeutic biopsy samples. As a result, peritumoral and Crohn's-like lymphoid reactions could not be evaluated. Nevertheless, we detected a strong association between CD8^+^ TILs and therapeutic response within our cohort.

Our study has several strengths. This study has investigated the association between therapeutic response and TILs within 134 pretherapeutic samples of rectal cancer, utilizing our prospectively maintained database, which integrates clinicopathological features, regimens of chemotherapy and radiation, tumor molecular markers, and immune-reaction status. This enabled us to test the association of lymphocyte and therapeutic response rigorously, controlling for potential confounders. In addition, we analysed not only lymphocytic density, but also the distribution and subtype in rectal-cancer tissue, allowing cell- and spatial-specific analyses.

## Conclusions

In conclusion, a higher CD8^+^ TIL density in rectal-cancer tissue is associated with better therapeutic response after neoadjuvant therapy, regardless of the regimens of radiation or chemotherapy. Our findings suggest a potential role for tumor-infiltrating CD8^+^ T-cells in promoting tumor response to radiation and chemotherapy. Further prospective studies are needed to validate these findings from the current hypothesis-generating study. Upon validation, these population-based data may have implications for selecting patients who are more likely to benefit from neoadjuvant therapy.

## Supplementary Material

goaa016_Supplementary_Data
