# Supplementary figures and images for: Density and distribution of lymphocytes in pretherapeutic rectal cancer and response to neoadjuvant therapy
Source: Gastroenterol Rep (Oxf). 2020 Jun 12;8(6):445–52. doi: 10.1093/gastro/goaa016 (PMC7793145; doi:10.1093/gastro/goaa016)

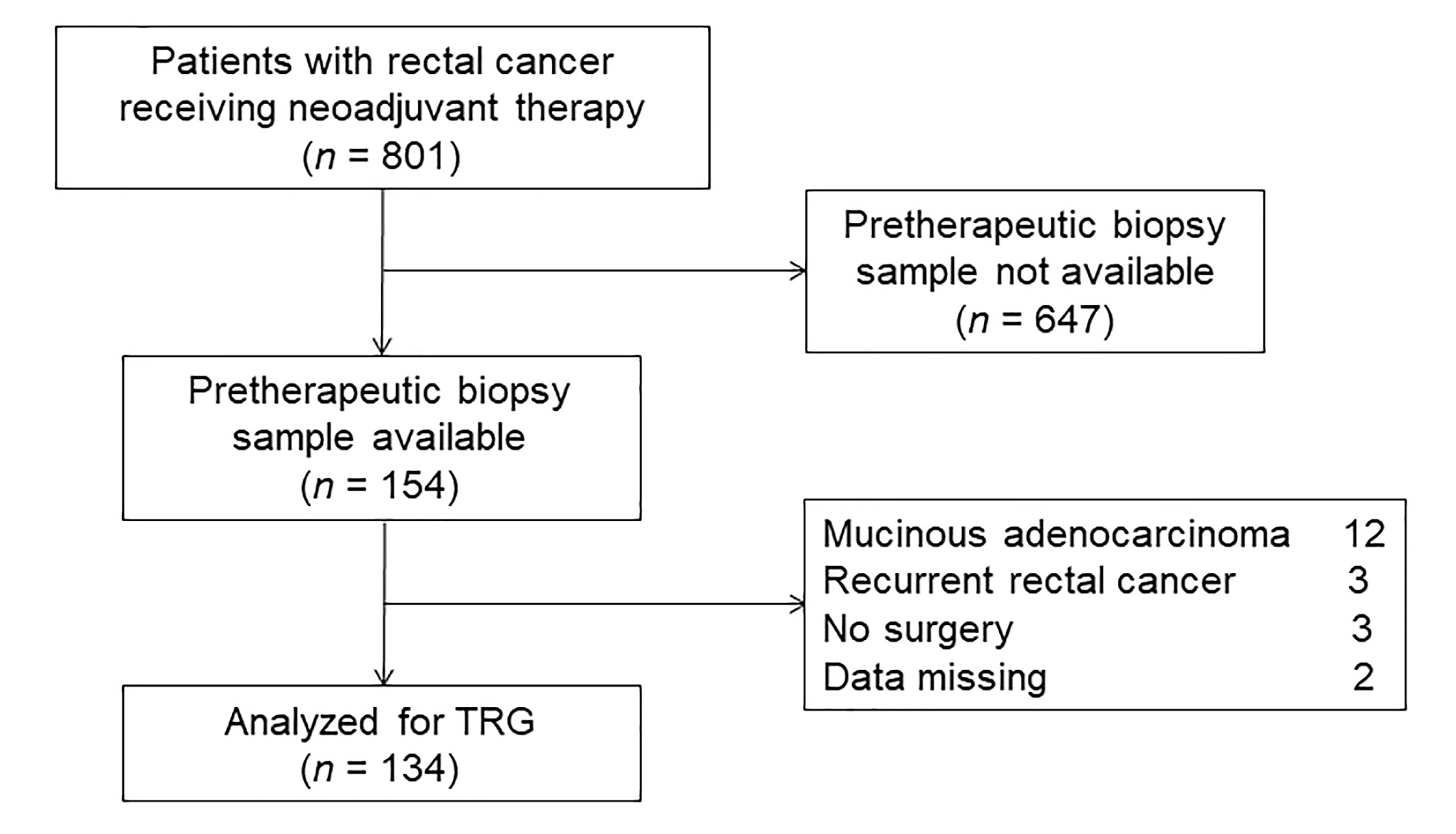

Supplement: goaa016_Supplementary_Data [file goaa016_supplementary_data.jpeg]
